# Trends of adverse drug reactions related-hospitalizations in Spain (2001-2006)

**DOI:** 10.1186/1472-6963-10-287

**Published:** 2010-10-13

**Authors:** Pilar Carrasco-Garrido, López Ana de Andrés, Valentín Hernández Barrera, Gil Ángel de Miguel, Rodrigo Jiménez-García

**Affiliations:** 1 Department of Preventive Medicine and Public Health, Rey Juan Carlos University, Alcorcón, Madrid. Spain

**Keywords:** ADR-related hospitalizations, Minimum Basic Data Set, Costs.

## Abstract

**Background:**

Adverse drug reactions (ADR) are a substantial cause of hospital admissions. We conducted a nationwide study to estimate the burden of hospital admissions for ADRs in Spain during a six-year period (2001-2006) along with the associated total health cost.

**Methods:**

Data were obtained from the national surveillance system for hospital data (Minimum Basic Data Set) maintained by the Ministry of Health and Consumer Affairs, and covering more than 95% of Spanish hospitals. From these admissions we selected all hospitalization that were code as drug-related (ICD-9-CM codes E), but intended forms of overdoses, errors in administration and therapeutics failure were excluded. The average number of hospitalizations per year, annual incidence of hospital admissions, average length of stay in the hospital, and case-fatality rate, were calculated.

**Results:**

During the 2001-2006 periods, the total number of hospitalized patients with ADR diagnosis was 350,835 subjects, 1.69% of all acute hospital admissions in Spain. The estimated incidence of admissions due to ADR decreased during the period 2001-2006 (p < 0.05). More than five percent of patients (n = 19,734) died during an ADR-related hospitalization. The drugs most commonly associated with ADR-related hospitalization were antineoplastic and immunosuppressive drugs (n = 75,760), adrenal cortical steroids (n = 47,539), anticoagulants (n = 26,546) and antibiotics (n = 22,144). The costs generated by patients in our study increased by 19.05% between 2001 and 2006.

**Conclusions:**

Approximately 1.69% of all acute hospital admissions were associated with ADRs. The rates were much higher for elderly patients. The total cost of ADR-related hospitalization to the Spanish health system is high and has increased between 2001 and 2006. ADRs are an important cause of admission, resulting in considerable use of national health system beds and a significant number of deaths.

## Background

In the current public health framework, the importance of drugs as determinants of the nation's health is an area that requires special attention [1,2], especially when we attempt to ascertain whether patterns of consumption reveal a rational use of medication.

Adverse drug reactions (ADR) are one of the leading causes of morbidity in developed countries and represent a substantial burden on health care resources [3]. Different studies carried out over the last 10 years to identify the incidence of adverse reactions associated with specific medications have revealed a significant number of hospital admissions (2.4%-6.5% of total hospital admissions [4-10], many of which are preventable. Therefore, this area of health surveillance is worthy of study.

Also to be taken into consideration are the high direct costs associated with the morbidity and mortality and treatment of ADRs. At the end of the 1990s, Bates et al calculated the annual costs generated by ADRs to be $5.6 million dollars [11], without taking into account associated indirect costs, such as time off work and reduced productivity [12,13].

We studied all hospital admissions and the associated health costs in Spain during the period 2001-2006 in order to estimate the incidence and characteristics of ADR-related hospitalization. This is a national study to be carried out in Spain to analyze ADR-related hospitalization

## Methods

A retrospective, descriptive, epidemiologic study was conducted, using the Minimum Basic Data Set (MBDS) as the data source. The MBDS is a national hospital admission database managed by the Ministry of Health and Social Policy that shows all hospitalizations for which the diagnoses are coded according to the Spanish version of the *International Classification of Diseases*, *Ninth Revision*, *Clinical Modification *(ICD-9-CM) [14,15]. The physician is the one responsible for making the report; it cannot be completed by another professional.

Aside from diagnoses at discharge (primary and secondary diagnosis), the variables covered by the MBDS are hospital data, patient data (name, date of birth, gender, place of residence, and date of admission), surgical and obstetric procedures, other procedures, and date and type of discharge. Estimated MBDS coverage is 95% of hospital admissions nationwide [16].

The criteria used for the identification of ADRs were those established by the World Health Organization. The WHO defines an ADR as a noxious and unintended response to a drug that occurs at doses normally used in humans for the prophylaxis, diagnosis, or therapy of disease, or for the modification of physiological function [17]. However, we must bear in mind that establishing the number of admissions due to ADRs depends significantly on the methodology used for their detection [18].

We selected MBDS data corresponding to hospital admissions during the study period with a diagnosis of ADR (ICD-9-CM codes E930 to E949; in any diagnostic position). Other adverse events (eg, accidents, suicides, accidental overdose, dosing errors) were excluded.

The data obtained included dates of admission and discharge, age, sex, average length of stay, and outcome (survival to hospital discharge or death).

We also calculated the average number of hospitalizations per year, annual incidence of hospital admissions (per 100,000 people), average length of stay in the hospital, and case-fatality rate (%). As the denominator, we used data on the population covered by hospitals included in the MBDS surveillance system, adjusted for population figures obtained from the 2001-2007 Spanish census projection. It was assumed that the age distribution of the population covered by these public hospitals was similar to that of the general population. The annual number of days of hospitalization was calculated by using the cumulative average number of cases per year and the average length of stay in the hospital.

We analyzed related comorbidity, mortality, and the direct medical cost to the health system of ADR-related hospitalizations. These costs were calculated using diagnosis related groups (DRG) for this condition. According to the DRG reimbursement system, every hospitalized patient belongs to a group of diagnostically homogeneous cases; therefore, patients within each category are similar clinically and are expected to use the same level of hospital resources. As a result, patients in the same DRG group are assigned the same reimbursement charges [19].

Differences in proportions were assessed using the Chi-square test, and confidence intervals (95% CI) were calculated. ANOVA was used for multiple comparisons. Statistical significance was set at p < 0.05 (two-tailed). Statistical analyses were performed using the Statistical Package for Social Sciences (SPSS for windows, version 14.0; Chicago, Illinois, USA). Multivariate analysis of time trends in study variables was performed using Poisson regression models. Values were deemed statistically significant at P < 0.05.

## Results

During the 2001-2006 period, a total of 350,835 individuals were hospitalized with an ADR as their primary or secondary diagnosis, according to the MBDS. The principal characteristics of the study population are summarized in Table 1. This figure represented 1.69% (95% CI, 1.65-1.73) of all acute hospital admissions in Spain.

**Table 1:** Principal characteristics of study population.

Characteristic	Value
**Population (n)**	350.835
	
**Sex (%, CI 95%)**	
Male	50.48 (47.18-53.78)
Female	49.52 (46.22-52.82)
**Age (%, CI 95%)**	
≤ 16	3.3 (2.9-3.6)
17-55	21.2 (20.9-21.4)
56-75	39.5 (39.2-39.7)
> 75	35.9 (35.6-36.1)
**Median duration of admission in days (interquartile range)**	8 (10)
**Died during admission (%-CI 95%)**	5.64 (5.32-5.96)
	

Minimum Basic Data Set. 2001-2006

In our study, 5.64% (95% CI, 5.32-5.96) of patients (n = 19,734) died during an ADR-related hospitalization, that is, 0.1% of all hospitalizations during the study period.

Table 2 shows the results obtained for the time trend in incidence of ADR-related hospitalizations using Poisson regression models adjusted by age and sex. The estimated incidence of admissions due to ADR decreased (145 cases per 100,000 people in 2001 to 136 cases per 100,000 people in 2006) (p < 0.05); median stay remained unchanged. Patients admitted with an ADR had a median stay of eight days (interquartile range 10).

**Table 2:** ADR- related hospitalizations.

Years	No. of patients with ADR-related hospitalization	Median stay (IQ)	Incidence*100,000	Total n° of patients MBDS	% ADR-related hospitalization MBDS
**2001**	58,804	8 (10)	145.93	3,297,074	1.78%
**2002**	60,632	8(10)	147.87	3,343,711	1.81%
**2003**	54,922	9(10)	131.19	3,444,.541	1.59%
**2004**	60,424	9(10)	142.73	3,496,238	1.73%
**2005**	56,072	9 (10)	129.71	3,541,107	1.58%
**2006**	59,981	8 (10)	136.89	3,589,728	1.67%
**Total**	**350,835**	**8 (10)**	**138.926**	**20,712,399**	**1.69%**

Minimum Basic Data Set: time trend in incidence using Poisson regression model, 2001-2006 (adjusted for age and sex): p < 0.05

For age group and sex, and during all the years of the study period, we observed that the proportion of ADR-related hospitalizations increased in both men and women aged 56 and over. More men than women were admitted, except in those aged > 75 years (42.5% [95% CI, 42.1-42.8]) (Table 3).

**Table 3:** Total ADR- related hospitalization by sex and age groups.

							
	**2001**	**2002**	**2003**	**2004**	**2005**	**2006**	**TOTAL**
	**% CI 95%**	**% CI 95%**	**% CI 95%**	**% CI 95%**	**% CI 95%**	**% CI 95%**	**% CI 95%**
**Male**							
≤ 16	4.1 (2.9-5.2)	4 (2.9-5.0)	3.7 (2.5-4.8)	3.7 (2.6-4.7)	2.9 (1.7-4.0)	3.1 (1.9-4.2)	3.6 (3.1-4.0)
17-55	25.6 (24.0-25.9)	24.4 (23.4-25.3)	23.6 (22.5-24.6)	22.2 (21.2-23.1)	19.6 (18.5-20.6)	19.5 (18.4-20.5)	22.5 (22.0-22.9)
56-75	45.6 (44.7-46.4)	45.9 (44.1-45.8)	44.8 (43.9-45.6)	44.3 (43.4-45.1)	43.6 (42.7-44.4)	42.4 (41.5-43.2)	44.3 (43.9-44.6)
> 75	24.5 (23.5-25.4)	26.4 (25.4-27.3)	27.7 (26.7-28.7)	29.7 (28.7-30.6)	33.7 (32.7-34.6)	34.9 (33.9-35.8)	29.5 (29.1-29.8)
**Female**							
≤ 16	3.4 (31.7-37.6)	3.6 (2.4-4.7)	3.1 (1.9-4.2)	2.9 (1.7-4.0)	2.3 (1.1-3.4)	2.4 (1.2-.35)	3 (2.5-3.4)
17-55	22.8 (21.8-23.8)	21.6 (20.6-22.6)	20.8 (19.7-21.8)	19.5 (18.4-20.5)	17.2 (16.1-18.2)	17.2 (16.1-18.2)	19.9 (19.4-20.3)
56-75	36.8 (35.8-37.7)	35.8 (34.8-36.7)	35.5 (34.5-36.4)	34.8 (33.8-35.7)	32.7 (31.7-33.6)	31.7 (30.7-32.6)	34.6 (34.2-34.9)
> 75	36.8 (35.8-37.7)	38.8 (37.9-39.6)	40.5 (39.5-41.4)	42.6 (41.7-43.4)	47.6 (46.7-48.4)	48.5 (47.6-49.3)	42.5 (42.1-42.8)
**TOTAL**							
≤ 16	3.7 (2.9-4.4)	3.8 (3.0-4.5)	3.4 (2.5-4.2)	3.3 (2.5-4.0)	2.6 (1.8-3.4)	2.7 (1.9-3.4)	3.3 (2.9-3.6)
17-55	24.2 (23.5-24.9)	23 (22.3-23.7)	22.2 (21.4-22.9)	20.9 (20.1-21.6)	18.4 (17.6-19.1)	18.4 (17.6-19.1)	21.2 (20.9-21.4)
56-75	41.2 (40.5-41.8)	40.5 (39.8-41.1)	40.2 (39.5-40.8)	39.6 (38.9-40.2)	38.2 (37.5-38.8)	37.1 (36.4-37.7)	39.5 (39.2-39.7)
> 75	30.7 (30.0-31.3)	32.5 (31.8-33.1)	34 (33.3-34.6)	36 (35.3-36.6)	40.6 (39.9-41.2)	41.7 (41.0-42.3)	35.9 (35.6-36.1)

Minimum Basic Data Set 2001-2006

Table 4 shows the diagnoses and medications most commonly associated with ADR. Antineoplastic and immunosuppressive drugs (n = 75,760), adrenal corticosteroids (n = 47,539), anticoagulants (n = 26,546), and antibiotics (n = 22,144) all appear within the first ten. The most commons diagnoses in cases of ADR-related hospitalization were neutropenia (unspecified) (5%), obstructive chronic bronchitis (4.9%), and congestive heart failure (3.1%).

**Table 4:** Drug group and diagnoses most frequently associated with ADR.

	ICD-9-CM code	Frequency	Percentage CI 95%
			
**Drug group**			
Antineoplastic and immunosuppressive drugs	E933.1	75,760	21.5 (21.2-21.7)
Adrenal cortical steroids	E932.0	47,539	13.5 (13.1-13.8)
Anticoagulants	E934.2	26,546	7.5 (7.1-7.8)
Cardiotonic glycosides	E942.1	24,662	7.0 (6.6-7.3)
Antibiotics	E930.8-E930.9	22,144	6.3 (5.9-6.6)
Diuretics	E944.4	21,818	6.2 (5.8-6.5)
Antirheumatics	E935.6	12,859	3.6 (3.2-.39)
Cardiac rhythm regulators	E942.0	11,943	3.4 (3.0-3.7)
Unspecified drug or medicinal substance	E947.9	11,134	3.2 (2.8-3.5)
**Diagnoses**			
Neutropenia, unspecified	288.0	17,803	5.0 (4.6-5.3)
Obstructive chronic bronchitis	491.21	17,239	4.9 (4.5-5.2)
Congestive heart failure	428.0	11,135	3.1 (2.7-3.4)
Encounter for chemotherapy and immunotherapy for neoplastic conditions	V58.1	10,840	3.0 (2.6-3.3)
Pneumonia	486	10,244	2.9 (2.5-3.2)
Diseases of respiratory system	519.8	6,951	1.9 (1.5-2.2)
Aplastic anemias	284.8	5,985	1.7 (1.3-2.0)
Other and unspecified adverse effect of drug	995.2	4,968	1.4 (1.0-1.7)
Acute renal failure	584.9	4,787	1.3 (0.9-1.6)
Toxic gastroenteritis and colitis	558.2	4,444	1.2 (0.8-1.5)

Minimum Basic Data Set, 2001-2006. International Classification of diseases (9^th ^Edition) - clinical Modification

Table 5 shows the development of costs for all ADR-related hospitalizations during the study period by age group and sex. Using the DRGs for the study codes, the estimated cost per patient and year was €3,857 in 2001 and €4,382 in 2006, with a total cost in 2001 of €226,128,029 and in 2006 of €272,971,610. The cost of admissions among these patients increased significantly (p < 0.05). If we eliminate the effect of the consumer price index, costs generated by patients in our study increased by 19.05% between 2001 and 2006.

**Table 5:** Hospitalization costs of the ADR-related hospitalizations according to sex and age group.

						
	**2001**	**2002**	**2003**	**2004**	**2005**	**2006**
						
**Male**						
≤ 16	4,160	4,876	5550	5,754	6,102	6,361
17-55	5,094	5,569	5995	5,915	5,916	6,059
56-75	3,874	4,270	4653	4,811	4,715	4,767
> 75	3,315	3,623	3883	4,097	4,017	4,009
**Female**						
≤ 16	4,270	4,631	5500	5,492	5,932	6,130
17-55	4,377	4,685	4919	5,162	4,940	4,979
56-75	3,665	3,992	4238	4,501	4,446	4,489
> 75	3,131	3,420	3676	3,932	3,865	3,870
						
**Total cost per patient and year (Euros)**	3,857	4,197	4,498	4,656	4,529	4,382
						
**Difference above consumer price index**	-----	220	146	32	- 271	-337

Minimum Basic Data Set, 2001-2006

For the whole study period, total hospital costs were greater for patients aged 56 years and over than in the other groups.

## Discussion

Our results show that there were 350,835 ADR-related hospitalizations (almost 1.7% of all hospitalizations) in Spain between 2001 and 2006. These results are similar to those obtained in Holland by Hooft et al, [4] who aimed to ascertain the incidence of ADR-related hospitalization over 1 year. However, in a population-based cohort study carried out in 2003, the same group found a prevalence of ADR-related admissions of 5.1% [7], which is consistent with the results reported by other authors, who obtained higher values of ADR-related hospitalization [5,6,10,20,21]. These differences are due to the different types of study and methodology used, as well as to the definition of ADR applied.

Since Lazarou et al. [22] concluded at the end of the 1990s that the incidence of fatal ADRs in US hospitals was extremely high (0.31% of all hospitalizations), other authors have reached similar conclusions a decade later [7]. Our results show that more than 5% of ADR-related hospitalizations had a fatal outcome (0.1% of all hospitalizations). These results are consistent with those obtained in a prospective study carried out in England by Pirmohamed [6] et al, who detected a 0.15% incidence of fatal ADRs.

The prevalence of ADRs varied between the different age groups, with elderly patients experiencing far more ADRs than children or adults. In our study the rates of ADR-related hospitalization have a frequency of 3.3% in children and a frequency of 35.9% in elderly (75 years and over). These results are consistent with data in the meta-analysis of Beijer HJ [23], who showed that the probability of being hospitalized due to ADR-related problems is 4 times higher for elderly people than for younger people (16.6% vs. 4.1%). Elderly patients are particularly vulnerable to ADRs because they take multiple-drug regimens and experience age-associated changes in pharmacokinetics and pharmacodynamics. Similarly, the Italian study by Onder et al [10] concluded that the most important determinant of risk for ADR-related hospital admissions in older patients is the number of drugs being taken (OR, 1.18; 95% CI, 1.11-1.25) for each new drug taken).

The most commonly involved drugs were antineoplastic drugs, and this is consistent with the results obtained by Zhang M et al.[24] using data from the Western Australia Hospital Morbidity Data System, which showed that medication is responsible for 11.0% of ADR-related hospital admissions in elderly Australians.

As was the case with other investigators, [4,7,9,25,26] we observed high percentages of ADRs were for anticoagulants and diuretics. These drug groups have a high innate toxicity, with both diuretics and oral anticoagulants requiring close monitoring for safe use. In addition, these drug groups are often used in elderly patients who are more susceptible to adverse reactions.

It is noteworthy that antibiotics appear in 22.1% of ADR-related hospitalizations in Spain. Similarly, research carried out in the United Kingdom by Kongkaew et al [5] in a systematic review of observational studies revealed that 42.6% of ADRs in children were associated with anti-infective drugs. In Italy, Trifiro et al. [8] studied the incidence of ADRs and ADR-related hospital admissions after visits to the emergency department and found that antibiotics were associated with 12.9% of ADRs. Should be noted the intrinsic value of such a coding system is easy to perform for drugs which have "clear" endpoints for toxicity (ADRs) like neutropenia (antineoplastic drugs/immunosuppressive drugs), diuretics (disturbances in electrolytes), adrenal corticoids (diabetes induction, osteoporosis induction etc.) and anticoagulants (bleeding).

In Spain, the total cost to the health system caused by ADR-related hospitalization during the study period was around €1.533 billion; annual costs increased from €226 million in the year 2001 to €272 million in 2006. This represents a considerable cost. It is interesting to note that recent studies have shown that the excess hospital cost of ADRs in the USA were estimated at $2000 to $3000 dollars per patient [11,12], which is somewhat lower than in our study.

Our study is limited in that the proportion of ADR-related hospitalizations is probably an underestimation of the real situation. With regard to underreporting, this can be attributed to different causes. Among these is the difficulty associated with the codification of these processes according to the International Classification of Diseases, Ninth Revision, which quite often does not include the drug causing the reaction, only the ADR without specifying the origin. Physicians might therefore consider this codification as to be used only for administrative purposes and would be less concerned with accurately recording these ICD codes. The minimum basic data set is a useful tool for the identification, quantification and analysis of adverse drug reactions, as has been demonstrated in previous studies [20]. However, according to data from the *Sistema Español de Farmacovigilancia *(Spanish Surveillance System), these reactions are clearly underreported in the hospital environment as they are little noted in discharge reports. We evaluated hospitalizations retrospectively and, therefore, depended on information from electronic medical records. In fact if we compare the results of our study with others conducted in Spain [27], in which the proportion of hospital admissions due to adverse reactions is 4.2%, it is three times higher than the result provided by this manuscript. This also occurs when comparing the results with those of other countries as the work of Lazarou et al. [22], or Van der Hooft et al [7].

Another limitation of this study may be that ADR-related hospitalizations also include cases in which the ADR occurred during the admission. Furthermore, hospitalizations for which the E-code corresponds to a secondary diagnosis may have been admissions during which the ADR occurred or admissions caused by the ADR.

The limitations of the MBDS as a tool for detecting ADRs primarily stem from the express mention of the corresponding ADR on the discharge report written by the physician, as only what is codified is transcribed, and the physician is the one responsible for making this report. A second limitation is the degree of codification of the diagnosis reported on the discharge report form: incorrect codification, variation among codifiers, human error. Physicians might therefore consider this codification as to be used only for administrative purposes and would be less concerned with accurately recording these ICD codes

Nevertheless, we believe that the length of the study period and the exhaustive data provided by the MBDS provide sufficient internal validity that, in quantitative terms, is seen in the constant frequency of episodes detected every year and, in qualitative terms, in the identification of age groups at greater risk. The high proportion of elderly patients and the cohort survival effect in women aged more than 70 years is consistent with the results of other studies.

Finally, the system of cost assignment was based on DRGs. Although DRGs have been used to improve the classification system for hospital cost analysis, they do present a series of limitations [28,29]. DRG tariffs were used as unit costs instead of real costs. Tariffs can introduce distortion for administrative reasons; in the case of death, the recognized tariffs are doubled. This system is not specifically designed for individual ADR-related hospitalisations.

The minimum basic data set is a useful tool for the identification, quantification and analysis of adverse drug reactions, as has been demonstrated in previous studies. However, according to data from the *Sistema Español de Farmacovigilancia *(Spanish Pharmacovigilance System), these reactions are clearly underreported in the hospital environment as they are little noted in discharge reports.

## Conclusions

During the study period, a total of 350,835 individuals were hospitalized due to ADRs in Spain (1.69% of all acute hospital admissions). The rates were much higher for elderly patients. The total cost of ADR-related hospitalization to the Spanish health system is high and has increased between 2001 and 2006.

In conclusion, ADRs are an important cause of admission, resulting in considerable use of national health system beds and a significant number of deaths. The risk is that with this data source, the prevalence or incidence of ADRs as a public health problem is underestimated.

## Competing interests

The authors declare that they have no competing interests.

## Authors' contributions

Pilar Carrasco-Garrido and Rodrigo Jiménez-García conceived of the study, have written the document, and supervised all aspect of its implementation.

Valentín Hernández-Barrera has analyzed the information, Ana López de Andrés and Ángel Gil de Miguel, helps with the reading and review.

All authors helped to conceptualize ideas, interpret findings, and review of the manuscript.

## Pre-publication history

The pre-publication history for this paper can be accessed here:

http://www.biomedcentral.com/1472-6963/10/287/prepub
